# Förster Resonance Energy Transfer (FRET) between Heterogeneously Distributed Probes: Application to Lipid Nanodomains and Pores

**DOI:** 10.3390/ijms131216141

**Published:** 2012-11-30

**Authors:** Radek Šachl, Lennart B.-Å. Johansson, Martin Hof

**Affiliations:** 1 Department of Biophysical Chemistry, Jaroslav Heyrovský Institute of Physical Chemistry, Academy of Sciences of the Czech Republic, Dolejškova 2155/3, Prague 8, 182 23, Czech Republic; E-Mail: radek.sachl@jh-inst.cas.cz; 2 Biophysical Chemistry, Department of Chemistry, Umeå University, S-901 87 Umeå, Sweden; E-Mail: lennart.johansson@chem.umu.se

**Keywords:** FRET, lipid domains, pores, lipid bilayer, rafts, fluorescent probes

## Abstract

The formation of membrane heterogeneities, e.g., lipid domains and pores, leads to a redistribution of donor (D) and acceptor (A) molecules according to their affinity to the structures formed and the remaining bilayer. If such changes sufficiently influence the Förster resonance energy transfer (FRET) efficiency, these changes can be further analyzed in terms of nanodomain/pore size. This paper is a continuation of previous work on this theme. In particular, it is demonstrated how FRET experiments should be planned and how data should be analyzed in order to achieve the best possible resolution. The limiting resolution of domains and pores are discussed simultaneously, in order to enable direct comparison. It appears that choice of suitable donor/acceptor pairs is the most crucial step in the design of experiments. For instance, it is recommended to use DA pairs, which exhibit an increased affinity to pores (*i.e.*, partition coefficients *K*_D,A_ > 10) for the determination of pore sizes with radii comparable to the Förster radius *R*_0_. On the other hand, donors and acceptors exhibiting a high affinity to different phases are better suited for the determination of domain sizes. The experimental setup where donors and acceptors are excluded from the domains/pores should be avoided.

## 1. Introduction

Liquid ordered L_o_ domains (referred to as rafts) [1] and pores [2] represent heterogeneities usually encountered in lipid bilayers. The lipid domains are usually small (10–200 nm), highly dynamic and are enriched in the presence of cholesterol and sphingomyelin [3]. The accumulation of cholesterol and sphingomyelin into the L_o_ domains could be ascribed to the existence of attractive forces between cholesterol and shingomyelin or between cholesterol and a saturated phosphatidylcholine [4]. The acyl chains are ordered in the domains, but molecules diffuse across the domains at a speed comparable to the remaining liquid-disordered L_d_ phase. The domains adopt a circular shape in order to minimize the line tension originating from the height mismatch between the L_o_ and L_d_ phases and the steric interactions at the phase interface [5]. In cells, the domains are connected with several functional roles, such as signaling, membrane trafficking or viral infection.

Model lipid bilayers represent a very simplified system, as compared to the plasma membrane of a cell. First of all, the plasma membrane has a more complex composition. This will influence the nature of the phase boundaries and probably decrease the line tension, which in turn may lead to the formation of smaller domains. Actually, domains of micrometer size were hitherto never observed in cell membranes. Also, cell membranes exhibit an asymmetric composition and are not studied under equilibrium conditions, as is the case with model lipid bilayers. Therefore, the nature of the domains in the model bilayer and in the plasma membrane is probably different.

Since the size of L_o_ domains is beyond the optical resolution of a conventional microscope [6,7], elaborate techniques, such as neutron scattering, stimulated emission depletion spectroscopy (STED) [8] or Förster resonance energy transfer (FRET) [9,10], are required for the size determination. However, solely the lastly named technique is able to determine domain/pore sizes smaller than 20 nm. Usually, the spectroscopic characterization of lipid domains is complicated by the fact that all lipid molecules partition to some extent into both the L_d_ and L_o_ phase. Nevertheless, as will be shown here, domain/pore sizes comparable to the Förster radius *R*_0_ can be estimated by FRET, despite a limited partitioning of the probes into the L_o_ phase. Here, the FRET analysis relies on a non-random distribution of donors (D:s) and acceptors (A:s) between domains/pores and the remaining bilayer surface. Actually, the best resolution can be expected if all D:s and/or A:s are excluded from the remaining bilayer surface. So far, this seems hypothetical, because few fluorophores exhibit the desirable property. For liquid-ordered (L_o_) domains, most probes are excluded from the densely packed region of ordered acyl chains. Cholera toxin labeled by Alexa 488 exhibits an increased affinity with a partition coefficients (*K*) ranging from 6 to 10 [7,11], depending on the lipid composition, as well as dipalmitoylphosphatidylethanolamine labeled by NBD with *K* = 4.3 [12]. The situation can be more favorable in the case of pores, which are usually built by bigger (as compared to the size of a fluorescent dye) protein/peptide molecules. Attachment of a dye to such a big molecule should not influence its partitioning, and the efficiency of pore labeling should thus only depend on whether the peptide/protein can also be found outside the pore.

In a typical qualitative FRET experiment, the FRET efficiencies of a DA pair localized in a bilayer, which does and does not contain membrane heterogeneities, are compared. This should preferentially be done by comparison of time-resolved fluorescence (TRF) decays for the two situations, but measurement of steady-state (SS) intensities is in principle possible as well. In order to determine domain/pore sizes, the measured TRF-decays must be fitted by an appropriate model, where the domain/pore radius is one of the optimized parameters. In this paper, it is assumed that the domains/pores have circular/cylindrical shape, respectively.

**Figure 1:**
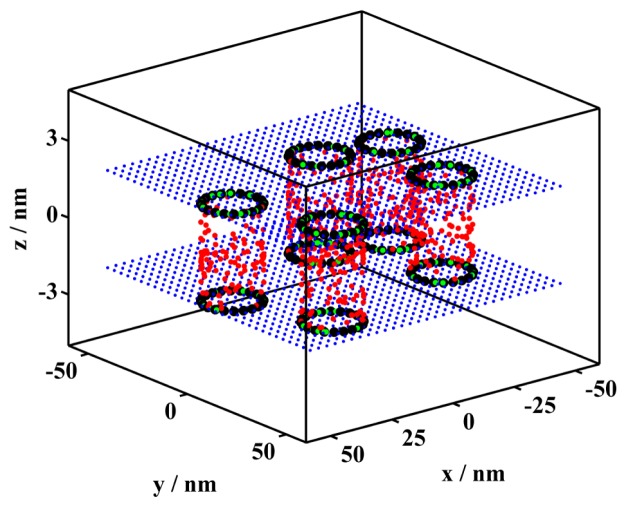
The replicated part of the lipid bilayer with randomly distributed toroidal pores. In the simulation, the shape of the pores has been approximated by a cylinder. The following extreme situation is assumed in the picture: The pore is formed by several peptide molecules, which exclusively reside in the pores. A certain peptide fraction is labeled by a fluorescent donor (green balls) in the polar end part. This means that donors are localized in the pores at the lipid/water interface only (*K*_D_ = ∞). Acceptors (red balls), on the other hand, have been distributed over the entire surface of the cylindrical pore (*K*_A_ = ∞). In order to highlight the pore-structure in the illustration, the pores have been heavily stained by the fluorophores.

The present paper is a continuation of our previous work [13] and aims at showing how FRET experiments should be performed and the data analyzed in order to achieve the best possible resolution in estimating nanodomain and pore sizes. On the contrary to the previous work [13], D:s and A:s are assumed to reside only at the lipid/water interface. Concerning pores, the donors are assumed to be distributed at the bottom/top part of the pores, while the acceptors are distributed over the entire pore surface (*cf.*Figure 1). Such an arrangement intends to mimic donors attached at a well defined position of a protein/peptide that forms toroidal pores, whereas the acceptor is a lipid analogue labeled in its headgroup. The present paper also reviews the Baumann-Fayer (B-F) model [14], which accounts for FRET between uniformly distributed probes in a homogenous bilayer. The B-F model establishes a basis for an extended modeling of heterogeneous distributions.

## 2. Results and Discussion

### 2.1. Limitations of FRET in the Detection of Nanodomains and Cylindrical Pores

It can be expected that the formation of domains/pores in the lipid bilayer disturbs a homogenous distribution of fluorescent probes. Under certain conditions, which are discussed throughout this paper, the appearance of these heterogeneities can be detected by Förster resonance energy transfer (FRET). This technique enables not only detection of membrane heterogeneities, but also a size determination of these structures. Sensitivity of FRET to the formation of membrane heterogeneities depends on how donors and acceptors are distributed between these structures and the remaining bilayer. In practice four situations are of interest: (1) The donors and acceptors are excluded from the domains/pores; (2) The donors and acceptors are preferentially localized within the domains/pores; (3) The donors prefer a localization within the domains/pores, while the acceptors are localized in the remaining bilayer; and (4) Acceptors are localized in the domains/pores and donors are excluded from them. Because the last situation gives similar FRET efficiencies as the third situation, it will not be discussed here. In general, the first two cases lead to increased FRET efficiencies (as compared to the uniform probe distribution), *i.e.*, to a faster fluorescence decay of donors and to the decrease in the SS-intensity. On the contrary, the last two cases result in the decrease of FRET efficiency, *i.e.*, in slower fluorescence decays and an increased SS-intensity. This happens due the formation of structures that segregate the donors from the acceptors. For discussing the limitations of FRET, it is convenient to introduce the ratio of steady-state (SS) intensities for the heterogeneous *I*_D_ and uniform *I*_D_(uni) distribution of probes. The ratio is formed by *I*_D_/*I*_D_(uni) when formation of domains/pores leads to decreased FRET efficiency or by *I*_D_(uni)/*I*_D_, respectively, when the energy transfer rate (=*ω*) is increased due to the formation of domains/pores. The ratio is formed in the way that it is always ≥ 1. According to our experience, domains are for ratio < 1.05 (red) beyond the resolution of TR-FRET and SS-FRET. For the range 1.05 < ratio < 1.1 (orange), the domains are close to the detection limit of TR-FRET, whereas the range 1.1 < ratio < 1.2 (yellow) represent domains that can be resolved by TR-FRET, but remain close to the detection limit of SS-FRET [13]. Finally, for the ratios > 1.2 (green), the domains can be detected by TR-FRET and SS-FRET.

The affinity of probes to the domains/pores and the remaining bilayer is characterized by the partition coefficient *K*_D,A_ = [probes inside]/[probes outside]). In the case of domains, the concentration is clearly calculated as the average number of probes in a domain, *N*_in_, divided by the domain area, π*R*^2^. Due to a large complexity of pores, it is not possible to find a general expression that would yield the entire pore surface for which donors and acceptors compete with other molecules. Therefore, it seems a good compromise to define [probes inside] in the simulation as *N*_in_/*V*_pore_, where *V*_pore_ = π*R*_pore_^2^*d* is the average volume of a pore. However, due to direct proportionality of this value to π*R*_pore_^2^ (since *d* is a constant) and due to resemblance of π*R*^2^ to π*R*_pore_^2^, it is more convenient to define [probes inside] as *N*_in_/π*R*_pore_^2^. As will be shown further in the text, this enables direct comparison between FRET diagrams for the domains and the rafts, respectively.

**Figure 2:**
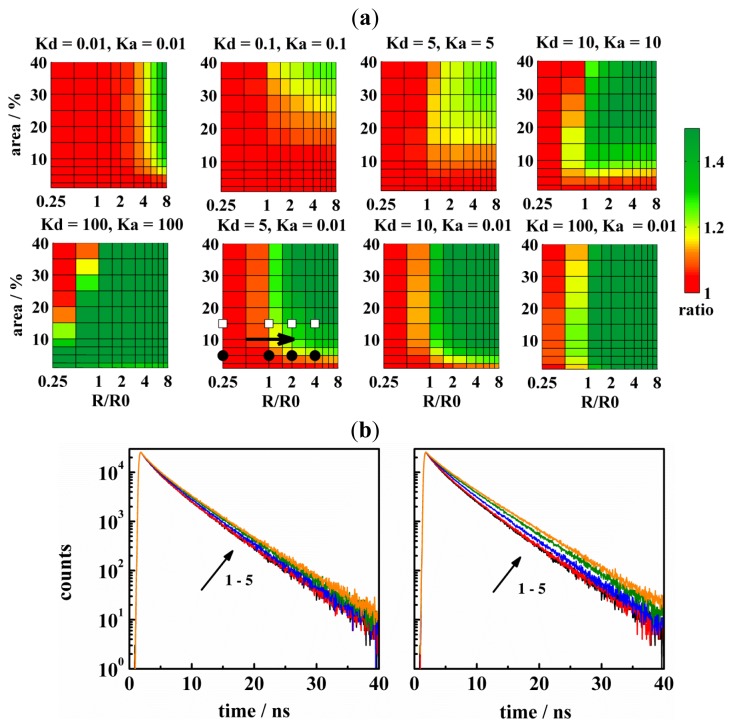
(**a**) The resolution of FRET in the determination of L_o_ domain sizes as a function of the domain area and the relative domain radius (*i.e.*, *R*/*R*_0_). The FRET resolution is given by the SS-ratio *I*_D_/*I*_D_(uni) or by the ratio *I*_D_(uni)/*I*_D_ (*cf.* the section MC simulations). A few points have been chosen from the diagrams and corresponding time-resolved fluorescence decays of donors displayed on the left of Figure 2b (filled circles) or on the right of Figure 2b (empty squares), respectively. The arrow indicates the order of TRF-decays in the part b of this figure. The D-lifetime was 6 ns in the L_o_ and L_d_ phase, and the probes were localized at the lipid water interface. Values exceeding 1.5 are displayed with the same color at the limiting value; (**b**) TRF-decays for a few chosen cases of Figure 2a. The order of the decays is indicated by the arrow and is the following: reference case for the uniform distribution of D:s and A:s in the homogenous bilayer (black), *R*/*R*_0_ = 0.25 (red), *R*/*R*_0_ = 1 (blue), *R*/*R*_0_ = 1 olive and *R*/*R*_0_ = 4 (orange). The area fraction of the domains in the bilayer, *A_r_*, was 5% (on the left, filled circles in Figure 2a) or 10% (on the right, empty squares in Figure 2a), respectively.

Situation 1: This situation is relevant when donors and acceptors are excluded from the domains/pores. Concerning the estimation of domain-sizes, this experimental setup appears attractive, because most probes exhibit an increased affinity to the liquid-disordered non-raft phase. However, as is illustrated in Figure 2a, FRET cannot resolve domains with radii comparable to the Förster radius *R*_0_, and domains occupying up to 40% of the entire bilayer area (tested region). The resolution improves with increasing domain size and fraction. The reason is that (i) domains larger than *R*_0_ make FRET across the domains impossible and (ii) higher amount of domains concentrates the acceptors in the non-raft phase.

**Figure 3:**
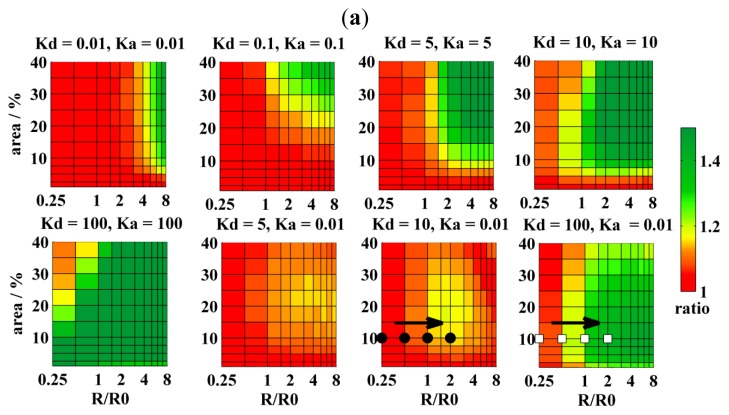

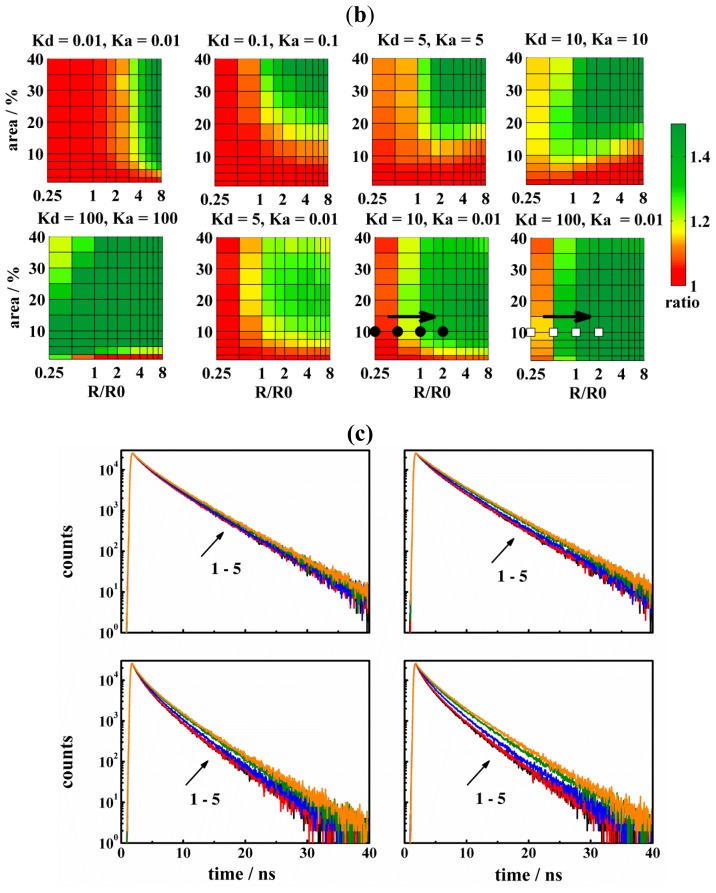
(**a**,**b**) The resolution of FRET in the determination of pore sizes as a function of the domain area and the relative domain radius (*i.e.*, *R*/*R*_0_). The FRET resolution is given by the SS-ratio. To enable direct comparison with the L_o_ domains, *K*_D_ and *K*_A_ have been kept the same. As in the previous case, a few points have been chosen from the diagrams and corresponding TRF-decays displayed in Figure 3c on the left (filled circles) or on the right (empty squares), respectively. Within the pores, donors were localized at the bottom/top part of the pores only (*cf.*Figure 1), whereas acceptors were distributed over the entire pore surface. The acceptor to lipid ratio was 1:200 in Figure 3a, while it was 1:100 in Figure 3(b). For more details, see also Figure 2a and Figure 1; (**c**) TRF-decays for a few chosen cases of Figure 3a (first row) or Figure 3a (second row), respectively. The order of the decays is indicated by the arrow and is the following: reference case for the uniform distribution of D:s and A:s in the homogenous bilayer (black), *R*/*R*_0_ = 0.25 (red), *R*/*R*_0_ = 0.5 (blue), *R*/*R*_0_ = 1 olive and *R*/*R*_0_ = 2 (orange). The area fraction of the domains in the bilayer, *A_r_*, was 10%. The left part of the figure displays four chosen decays of Figure 3a (filled circles), where *K*_D_ = 10 and *K*_A_ = 0.01, whereas the right part of the figure shows four chosen decays of Figure 3a, b (empty squares), where *K*_D_ = 100 and *K*_A_ = 0.01.

Regarding pores and their structure, two main types exist [2,15]. Toroidal pores exhibit high curvature made up by peptides and lipids. For example, these include antibacterial peptides, such as magainin-2 [16,17]. On the other hand, barrel-stave pores are exclusively formed by peptides (e.g., alamethicin [18]), and the bilayer does not bend in the pores [19]. Pores may also form proteins, such as Bax [20,21], or a bacterial toxin α-hemolysin [22], but the pore structure is less known. Situation 1 seems thus convenient for barrel-stave pores, which do not offer any free area for the lipid-based probes, as well as for toroidal pores, where it is to expect that *K*_D,A_ of cylindrically shaped probes would not exceed unity due to high pore-curvature. As is shown in Figure 3a, resolution of pore-sizes comparable to *R*_0_ is as poor as in the case of domains and improves a little with increasing the pore radius and the area fraction. Naturally, resolution can be further improved by using higher acceptor concentrations (*cf.*Figure 3a,b with acceptor to lipid ratios 1:200 or 1:100, respectively).

**Figure 4:**
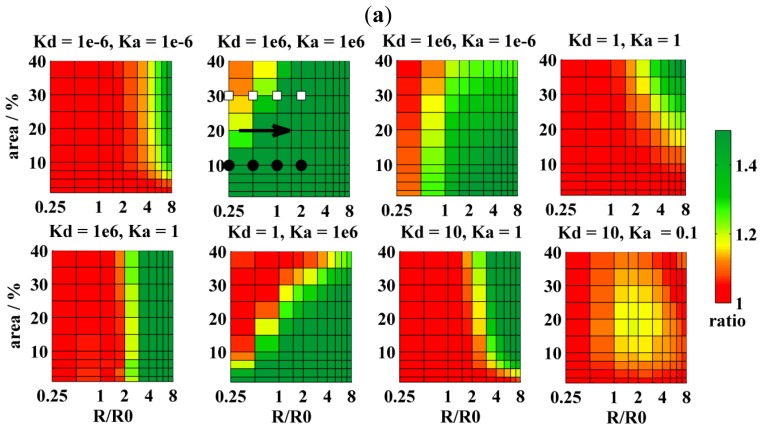

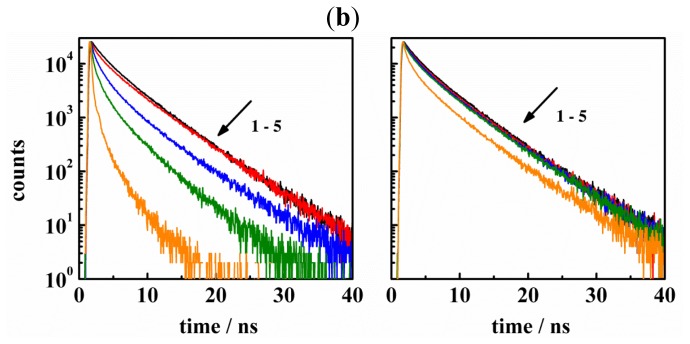
(**a**) The resolution of FRET in the determination of pore sizes for cases where donors have increased affinity to pores as a function of the domain area and the relative domain radius (*i.e.*, *R*/*R*_0_). The FRET resolution is given by the SS-ratio. As in the two previous cases, a few points have been chosen from the diagrams and corresponding TRF-decays displayed in Figure 2b on the left (filled circles) or on the right (empty squares), respectively. Within the pores, donors were localized at the bottom/top part of the pores only (*cf.*Figure 1), whereas acceptors were distributed over the entire pore surface. For more details, see also Figure 2a and Figure 1; (**b**) TRF-decays for a few chosen cases of Figure 4a. The order of the decays is indicated by the arrow and is the following: reference case for the uniform distribution of D:s and A:s in the homogenous bilayer (black); *R*/*R*_0_= 0.25 (red); *R*/*R*_0_= 0.5 (blue); *R*/*R*_0_= 1 olive; and *R*/*R*_0_= 2 (orange). The area fraction of the domains in the bilayer, *A_r_*, was 10% (on the left, filled circles in Figure 2a) or 30% (on the right, empty squares in Figure 2a), respectively.

In the Situation 2 donors and acceptors have an increased affinity to domains/pores. This experimental setup leads to a closer contact between donors and acceptors, even for small domains/pores covering a low area fraction. Therefore, this is better suited for the determination of domain/pore sizes that are comparable to *R*_0_. For instance, structures with *R* ≈ *R*_0_ and occupying 10% of the entire bilayer area are resolvable by D/A pairs with *K*_D,A_ = 10. Utilization of probes with *K*_D,A_ = 5 will shrink the resolvable range of *R* and *A_r_* to still experimentally interesting values 2 < *R*/*R*_0_ < 8 and 15 < *A_r_* < 40 (Figure 2a, Figure 3a–c and Figure 4a,b). It is evident from the figures shown that situation 2 is more convenient in the determination of pores rather than domain sizes for the same combination of *K*_D,A_ values, *i.e.*, for the same number of probes within domains/pores at the given *R*. This is also true regarding the availability of suitable fluorescent probes. Available probes exhibit only modest affinity to the L_o_ domains with the highest known value of *K* = 6 for Cholera toxin labeled by Alexa 488 [7,11]. Most probes are excluded from L_o_ domains, mainly due to more packed environment of L_o_ phase, as compared to the L_d_ phase [23].

Attachment of a dye to a peptide/protein that builds the pore appears as the best solution for preferential labeling of the pores. Theoretically, if all peptide/protein molecules formed the pores, 100% labeling efficiency of pores could be achieved. Such extreme cases have been simulated as well and are displayed in Figure 4a,b. The best resolution is practically achieved when *K*_D,A_ reaches 100 (compare Figures 4a and 3b). Such a high partition coefficient results in a dramatic change of the decay (with respect to the uniform distribution of probes in the homogenous bilayer) and allows for detection of pore sizes as small as 0.25 *R*_0_ and occupying a few percent of the bilayer area. The only region where the resolution is poor has been found for small domains/pores (*R* ≈ 0.5*R*_0_), which occupy more than 30% of the bilayer area. If the pores were formed by six peptide/protein molecules and these were exclusively found in the pores, such conditions would correspond to a rather high peptide-to-lipid ratio of 1:30. Despite this limitation, situation 2 remains as the best choice in the determination of pore sizes.

In the Situation 3, it is assumed that donors have increased affinity to the domains/pores, whereas acceptors are excluded from them. This situation appears as a reasonable compromise between the availability of fluorescent probes and the resolution of FRET. Recently, we have shown [7] that a DA pair represented by CTxB labeled by Alexa 488 (*K*_D_ = 6) and DiD (*K*_A_ = 0.01–0.005) could determine domains with *R* = 8 nm and *A_r_* = 6% at a relatively high acceptor to lipid ratio 1:80. In order to detect domains comparable to *R*_0,_ the ratio 1:100 is recommended, but it can further be reduced to 1:200 if probes with *K*_D_ = 10 are used (*cf.*Figure 2a). Since the pore interior must remain empty (*cf.*Figure 1, donors which reside in the pores cannot be as efficiently separated from the acceptors as in the case of domains. This is why situation 3 is less useful for the determination of pore sizes at the same combination of *K*_D_ and *K*_A_ (compare Figures 2a and 3a). In this case, *K*_D_ > 100 or the acceptor-to-lipid ratio of 1:100 are recommended, respectively.

The fourth and last situation would arise when at least a member of the DA pair had the same affinity to the domains/rafts and the remaining bilayer surface. In the extreme case, probes are uniformly distributed as in the homogenous bilayer (*K*_D_ = 1 and *K*_A_ = 1), and consequently no domains can be detected. On the other hand, such unfavorable conditions should still enable the detection of pores, because FRET efficiency for probes uniformly distributed between the pores and the remaining bilayer must at certain *R* and *A_r_* start deviating from the uniform distribution of probes in the homogenous bilayer. The difference allows for the determination of pore sizes when *R*/*R*_0_ > 4 and *A_r_* > 30. This limiting case would correspond to the protein/peptide-to-lipid ratio of 1: 578 if the pores were built by six protein/peptide molecules [6] and the peptide/protein worked as a donor/acceptor as well (see also above). A slightly better resolution is achieved for *K*_D_ = 10^6^ and *K*_A_ = 1 (*cf.*Figure 4a). In this case, donors are efficiently concentrated into the pores, while acceptors are uniformly distributed between the pores and the remaining bilayer. This causes a partial separation of donors from the acceptors and decreased FRET efficiency. On the other hand, a seemingly identical situation characterized by *K*_D_ = 1 and *K*_A_ = 10^6^ leads to a significant increase in FRET efficiency, enabling the determination of either small pores (*R* ≈ *R*_0_) occupying only a few percent of the bilayer area, or larger pores (*R* > 2*R*_0_) covering several tens of percent of the bilayer area (*cf.*Figure 4a). Formation of L_o_ domains leads to an increased bilayer thickness and to a decreased lipid headgroup area. Risselada *et al*. [24] report, for instance, that the bilayer thickness increases from 4.0 nm to 4.6 nm and that the headgroup area decreases from 0.58 nm^2^ to 0.4 nm^2^ when going from the L_d_ to the L_o_ phase. These two phenomena have an opposite effect on the FRET efficiency. A reasonable quantitative estimate can be done by means of the B-F model, which accounts for FRET occurring in a homogenous lipid bilayer. Increase of *d* of about 10% or 20% results in the increase of the steady-state ratio from 1 to 1.035 or 1.073, respectively. On the other hand, shrinking of the average headgroup area of about 6% (which corresponds to the domain area fraction of 20%) results in the decrease of the ratio from 1 to 0.965. Because both effects are small and compensate each other, the bilayer thickness and lipid headgroup area were assumed to be constant in the simulations.

### 2.2. Searching for the Size of the Liquid-Ordered Domains

In our approach, the estimation of domain sizes is based on fitting the computed decay curve provided by MC simulations to the experimental one. Shape of the decay depends on the following parameters, which can be kept either free or fixed during the optimization routine: the donor fluorescence decay [*F*(*t*)]; the Förster radius, the bilayer thickness (*d*); the partition coefficients of donors (*K*_D_) and acceptors (*K*_A_); the reduced surface concentration of acceptors (*C*_A_); the domain radius (*R*); as well as the area fraction occupied by the domains in the bilayer (*A_r_*). In order to decrease the number of local minima, the number of optimized parameters is reduced as much as possible. For instance, the output parameter of the domain radius was rather insensitive to changes of the input parameters *K*_D_ and *K*_A,_ as well as to moderate changes of *C*_A_ in DOPC (49%)/Chol (25%)/Sph (19%)/DOPG (5%)/G_M1_ (2%) bilayer (*cf.* the list of abbreviations) containing small amounts of Cholera toxin (CTxB) [7]. By using the correct input values of *K*_D_ = 6 and *K*_A_ = 0.01, the domain radius found was 8 nm, and the domains occupy 6% of the bilayer surface. Interestingly, we have observed that reduction of *K*_D_ from six to three shifted the position of the chi-squared minimum to *R* = 8 nm and *A_r_* = 12%, while the increase of *K*_D_ to 9 resulted in *R* = 7 nm and *A_r_* = 5 % (*cf.*Table 1). A variation of *K*_A_ in the range ± 0.002 has not affected the shape of the decay, while a change of *C*_A_ in the range ±10% had a negligible impact on both *R* and *A_r_*. A low sensitivity of *R* to *K*_D_, *K*_A_ and *C*_A_ may open new possibilities and advantages for the data analysis: due to experimental difficulties to measure partition coefficients at the point of the phase diagram where the domains are not visible by a microscope, *K*_D_ and *K*_A_ can be measured at closest points of the phase diagram where the bilayer is microscopically phase separated and used at the point of interest as well. Values of *K*_D_ and *K*_A_ do not necessarily need to be the same as at the original point, but due to the low impact of partition coefficients on *R*, values of *K*_D_ and *K*_A_ being lower or higher than the true ones are not expected to influence the *R* estimation substantially. Similarly, reduced surface concentration *C*_A_ can be estimated for the non-separated homogenous lipid bilayer by means of the Baumann-Fayer model (see the section above) and used at the point of interest. By this approach, the number of optimized parameters could be reduced to only two, *i.e.*, the domain radius (*R*) and the area fraction of domains (*A_r_*) occupying a bilayer.

## 3. Experimental Section

### 3.1. FRET Modeling of Uniformly Distributed Probes

Energy transfer/migration within an ensemble of fluorescent molecules is usually described within a two-particle approximation [14]. It assumes an independent pairwise dipole-dipole interaction between the excited donor and any acceptor. In DDEM, the excitation energy reversibly jumps from an excited to an unexcited donor. The two-particle approximation, however, excludes transfer steps via the previously unexcited molecule further to another unexcited donor. For describing FRET in a system where the excited energy can be transferred to more than one acceptor, it is convenient to introduce a function *G*(*t*), which describes the probability that the initially excited donor is still excited at a time *t* later. Then the decay *F*(*t*) of a donor surrounded by acceptors is given by [14]:

(1) $$F(t)=G_{\text{DA}}(t)\sum_{i}\alpha_{i}\text{exp }(-t/\tau_{i})$$

where ∑*_i_α_i_*exp(−*t*/*τ_i_*) represents the decay of a donor in the absence of FRET. By summing up over all pairwise interactions exp(−*λω_i_*(*r_i_*)*t*) in a lattice, where each lattice site is occupied by an acceptor with the probability *p*, one obtains a general expression that accounts for energy transfer/migration between uniformly distributed donors and acceptors in systems with unspecified geometry. See [14,25] for the derivation.

(2) $$\text{ln }G_{\text{DA}}(t)=-\frac{\rho }{\lambda }\int_{0}^{\infty }(1-e^{-\lambda \omega (r)t})u(r)dr$$

Here, *λ* is a constant equal to 1 for FRET and to 2 for DDEM, respectively, *ω* is the energy transfer rate and *ρ* is the number density of acceptors. *u*(*r*) denotes a homogenous spatial distribution of donor-acceptor distances (*r*). The dynamic limit means that the reorienting rate of the interacting molecules is much faster than an energy transfer event. Hence, *ω* can be averaged before any transfer occurs and is therefore time-independent. For an ensemble of donors and acceptors, the dynamic limit is a reasonable approximation, provided that an excited donor may interact with many acceptors, which represent a wide range of orientations at the instant of the transfer.

**Figure 5:**
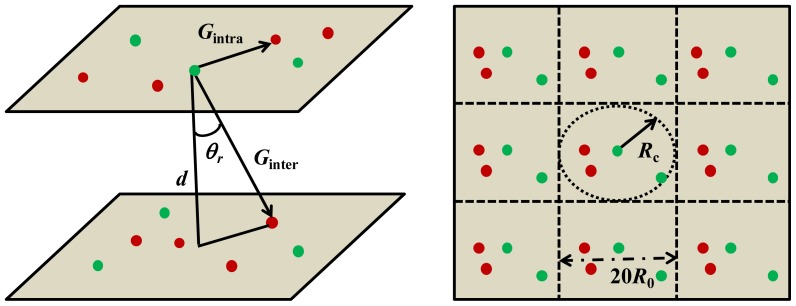
**(left)** Intra- and inter-energy transfer from donors (green spheres) to acceptors (red spheres) in a lipid bilayer. *d* denotes the bilayer thickness and *θ*_r_ the angle between the bilayer normal and the vector connecting D/A. (**right**) A schematic top view of the lipid bilayer divided into a replicated box (in the middle) and nine neighboring replicas. The size of the replicated box is 20*R*_0_. Energy transfer rate of the acceptors that are found outside the cut-off radius *R*_c_ = 10*R*_0_ is calculated via the continuous approach.

In a lipid bilayer the excitation energy can be transferred either to a molecule localized in the same or the opposite leaflet (Figure 5). These processes are referred to as *intra*-FRET [*G*_intra_(*t*)], and *inter*-FRET [*G*_inter_(*t*)], respectively. The total survival probability *G*_tot_(*t*) is given by the joint probability according to:

(3) $$G_{\text{tot}}(t)=\prod_{j}G_{j,\text{inter}/\text{intra}}(t)$$

when FRET/DDEM occurs in the same plane the distribution function *u*(*r*) = 2π*r*. The integration of Equation 2 yields the final expression for *G*_intra_(*t*) [14]:

(4) $$\text{ln }G_{\text{intra}}(t)=-C_{2}\lambda^{-2/3}\Gamma \left(\frac{2}{3}\right)\;{\left(\frac{t}{\tau }\right)}^{1/3}$$

In Equation 4,*C*_2_ denotes a reduced surface concentration and represents the average number of acceptor molecules within an area π*R*_0_^2^ surrounding a donor. *Γ* is the gamma function.

Similarly, one can obtain the analytical expression for the survival probability *G*_inter_[14]:

(5) $$\text{ln }G_{\text{inter}}(t)=-\frac{C_{2}}{3}{\left(\frac{d}{R_{0}}\right)}^{2}{\left(\frac{2\nu }{3}\right)}^{1/3}\int_{0}^{\langle \kappa^{2}\rangle \nu }(1-e^{-s})s^{-4/3}ds$$

In this expression, $s=\frac{3}{2}\lambda {\left(\frac{R_{0}}{d}\right)}^{6}{\text{cos}}^{6}\;\theta_{r}\langle \kappa^{2}\rangle \frac{t}{\tau }\text{and}=\lambda {\left(\frac{R_{0}}{d}\right)}^{6}\frac{t}{\tau }$. The distance between the bilayer sheets (*d*) approximately corresponds to the bilayer thickness, the angel (*θ*) is between the bilayer normal and the vector connecting the donor and the acceptor (Figure 5) and 〈*κ*^2^〉 is the mean-square average of the angular part of the dipole-dipole interaction.

### 3.2. Handling the Lack of Analytical Models for Heterogeneous Probe Distributions

Donors solubilized in a lipid bilayer that contains domains/pores are surrounded by unique density distributions of acceptors. This complicates the derivation of an analytical model that would account for FRET/DDEM processes. Despite this complication, several approaches relying on utilization of analytical equations have been developed and were described in a recent review [26]. So far, the most frequently used approach assumes an infinite phase separation [26,27], where FRET across the phase separation boundary is not considered. In this case, TRF-decays can be analyzed by means of the Bauman-Fayer (B-F) model (*cf.* Equations 4 and 5). This yields the acceptor surface concentrations *C*_2A_ and *C*_2B_ in phases A and B. These are related to the partition coefficient of the acceptors *K*_A_ = *C*_2A_/*C*_2B_. Not surprisingly, *K*_A_ values estimated by B-F model deviate from the true partition coefficient with decreasing domain size. Because the true partition coefficient also can be obtained by using other methods, the differently obtained *K*_A_ values can be compared and the domain size can be estimated. The limitations following from the use of an analytical model can be circumvented by a numerical analysis of the decays.

#### 3.2.1. Monte Carlo Simulations

A typical run of the MC simulation will be exemplified on the phase separated bilayer containing liquid-ordered domains. Small modifications are necessary in applications to a bilayer containing cylindrical pores. The following steps are usually carried out: (i) A certain random number of circular domains with uniform size is generated. The mean number of domains corresponds to the predefined area fraction the domains occupy in the bilayer; (ii) the donors and acceptors are generated to exhibit a certain probability of localization within and outside the domains. The distribution is described by an equilibrium constant *K*_D,A_ (= [probes within L_o_ domains]/[probes outside L_o_ domains]); (iii) A donor is randomly excited and its excitation energy is transferred to an acceptor. The time for this event to occur depends on the overall energy transfer rate *Ω_i_* according to [28,29]:

(7) $$\Delta t_{i}=-\text{ln }\alpha /\Omega_{i}$$

where *α* denotes a random number between 0 and 1. The total energy transfer rate is calculated as a sum of energy transfer rates from the excited donor *i* to all acceptors (first term of Equation 7). Acceptors that are beyond the cut-off distance 10*R*_0_ are included via the continuum approach (second term of Equation 7) [28]:

(8) $$\Omega_{i}=\sum_{j}\frac{3}{2}\kappa_{ij}^{2}{\left(\frac{R_{0}}{R_{ij}}\right)}^{6}\tau_{\text{D}}^{-1}+C_{2}{\left(\frac{R_{0}}{R_{c}}\right)}^{4}\tau_{\text{D}}^{-1}$$

Here *j* (1 ≤ *j* ≤ *N*) refers to the number of acceptors (*N*) found within a cut-off distance *R*_c_. *R*_ij_ is the distance between the i-th donor and j-th acceptor. Each generated configuration set is used 100 times in the calculation step before a new configuration set is generated. In order to assure for a good statistics, new configurations are usually generated 3000 times. Periodic boundary conditions with the size of the replicated box of 20*R*_0_ × 20*R*_0_ mimic an infinitely large membrane. The simulations correspond to energy transfer taking place under the dynamic limit condition and among isotropically oriented donors and acceptors. Therefore, kappa factor <*κ*_ij_^2^> = 2/3. The outcome of each simulation step is the time interval Δ*t*_i_ between the excitation and energy transfer event. By constructing a histogram made up by the number Δ*t*_i_ intervals belonging to each channel, *G*(*t*) function is obtained and the decay of donors undergoing FRET calculated (*cf.* Equation 1).

## 4. Conclusions

This paper demonstrates that the liquid-ordered nanodomains and pores with radii of a few nanometers and occupying several percent of the entire lipid bilayer can be determined by Förster resonance energy transfer combined with Monte Carlo simulations. The FRET resolution mainly depends on the affinity of donors and acceptors to the domains/pores. Therefore, to reach the best possible resolution choice of a suitable donor/acceptor pair is a most crucial step in the design of an experiment. When both donors and acceptors are preferentially localized in the pores appears to be the best experimental setup for the determination of pore sizes. This could for example be achieved by attaching a probe to a peptide/protein molecule, which usually forms the pore. The same experimental setup would also be convenient for the determination of domain sizes. But because probes with high affinity to the liquid-ordered domains are still missing, it is recommended to use a DA pair with high affinity to different phases instead. For instance, a pair with realistic partition coefficients of *K*_D_ = 6 and *K*_A_ = 0.01 resolves a broad range of domain sizes. Although majority of probes is excluded from the domains/rafts or is evenly distributed between these structures and the remaining bilayer, the case when the DA pair is excluded from the structures is not recommended.

## Figures and Tables

**Table 1:** The table shows to which extent the input parameters of the partition coefficient for donors *K*_D_ and acceptors *K*_A_ and the acceptor surface concentration *C*_A_ influence the optimized values of the domain radius, *R*, and the area fraction of the domains, *A_r_*. The real domain sizes are shown in the first row marked as “reference”.

	*C*_A_	*K*_D_	*K*_A_	*R*/nm	*A*_r_/%
reference	1.52	6	0.01	8	6
*K*_D_ 50% higher	1.52	6 + 3	0.01	7	5
*K*_D_ 50% lower	1.52	6 − 3	0.01	8	12
*C*_A_ 10% higher	1.52 + 0.15	6	0.01	8	8
*C*_A_ 10% lower	1.52 − 0.15	6	0.01	7	7

## Abbreviations

*A_r_*: area fraction of the L_o_ domains
B-F: Baumann-Fayer
*C*_2_: reduced surface concentration
Chol: cholesterol
CTxB: cholera toxin
DDEM: donor-donor energy migration
DOPC: dioleoylphosphatidylcholine
DOPG: dioleoylphosphatidylglycerol
FRET: Förster resonance energy transfer
*G*^s^(*t*): the excitation probability of the initially excited donor
G_M1_: monosialotetrahexosylganglioside
MC: Monte Carlo
L_d_: liquid disordered phase
L_o_: liquid ordered phase
*K**_i_* (*i* = D or A): partition coefficient of donors (*i* = D) or acceptors (*i* = A)
*R*: radius of a lipid domain or a pore
*R*_pore_: radius of a pore
*R*_0_: Förster radius
SS: steady-state
Sph: sphingomyelin
TRF: time-resolved fluorescence
*κ*: the angular dependence of dipole-dipole coupling
*τ*_D_: the fluorescence lifetime of the donor
